# Epidemiological models and COVID-19: a comparative view

**DOI:** 10.1007/s40656-021-00457-9

**Published:** 2021-08-25

**Authors:** Valeriano Iranzo, Saúl Pérez-González

**Affiliations:** 1 Department of Philosophy, University of Valencia, Valencia, Spain; 2 Center for Logic, Language, and Cognition (LLC), Department of Philosophy and Education Sciences, University of Turin, Turin, Italy

**Keywords:** Agent-based models, Compartmental models, COVID-19, Decision-making, Epidemiology, Prediction

## Abstract

Epidemiological models have played a central role in the COVID-19 pandemic, particularly when urgent decisions were required and available evidence was sparse. They have been used to predict the evolution of the disease and to inform policy-making. In this paper, we address two kinds of epidemiological models widely used in the pandemic, namely, compartmental models and agent-based models. After describing their essentials—some real examples are invoked—we discuss their main strengths and weaknesses. Then, on the basis of this analysis, we make a comparison between their respective merits concerning three different goals: prediction, explanation, and intervention. We argue that there are general considerations which could favour any of those sorts of models for obtaining the aforementioned goals. We conclude, however, that preference for particular models must be grounded case-by-case since additional contextual factors, as the peculiarities of the target population and the aims and expectations of policy-makers, cannot be overlooked.

## Introduction

Mathematical models are key tools in many scientific fields (Magnani & Casadio, 2016; Weisberg, 2013). Researchers use models to represent and study diverse kinds of systems (e.g., cells, planets, social networks, etc.). Models help them to learn about target systems, explain observed phenomena, organise knowledge, develop concepts, etc. (Bokulich, 2011; Knuuttila, 2011; Nersessian, 2010). Nonetheless, some concerns and doubts have been raised regarding the relevance of mathematical models for medical practice and health interventions (see, for instance, Ioannidis et al., 2020). Those worries are usually raised by advocates of the Evidence-Based Medicine (EBM) approach, according to which (clinical and policy) decision-making should be guided by the best available evidence (Thompson, 2010).

During the COVID-19 pandemic, maybe even more clearly than in previous health crises, epidemiological models have played a crucial role (Eubank et al., 2020; Holmdahl & Buckee, 2020; Rhodes et al., 2020). They have been used to predict the evolution of the pandemic, to estimate the effect of health interventions, to anticipate side effects, etc. Furthermore, given the lack of other decision-support tools (e.g., high-quality experimental evidence), especially in the first stages of the pandemic, models became the main guide for political decision-making (McBryde et al., 2020). In the United Kingdom, for instance, the government’s approach to the pandemic was mainly informed by epidemiological models run at the Imperial College London. In this sense, an update to a model related with intensive-care unit (ICU) bed occupancy (from 15 to 30% of hospital cases), which had a great impact on the number of predicted deaths, was highly relevant to the implementation of the first lockdown on 23th March (Adam, 2020). Similarly, in France, the scientific committee formed to inform public decision in the handling of the COVID-19 pandemic took epidemiological models as the main reference (Manzo, 2020). The judgements and recommendations released by this committee in mid-March led the French government to introduce several measures to reduce social interaction.

The aim of this paper is twofold. Firstly, to characterize the epidemiological models invoked during the COVID-19 crisis. We will discuss the so-called “theory-driven models”, particularly those kinds most widely used, that is, compartmental models and agent-based models (ABMs).^1^ Our second goal is to assess the theoretical strengths and weaknesses of those models. Factors such as the urgency to provide a guide for policy-making or the quality of the available data may limit the empirical success of models. But it should be made clear that here we are not concerned about the actual performance of particular models. Rather, we will focus on their general features and analyse how they can contribute to turn epidemiological models into effective tools for both prediction and policy-making. According to this, Sect. 2 introduces compartmental models (Sect. 2.1) and accounts for their capacities and limitations (Sect. 2.2). Section 3 deals with ABMs. It presents their main traits (Sect. 3.1), highlights some remarkable advantages (Sect. 3.2), and identifies certain problematic aspects (Sect. 3.3). Section 4 scrutinises how compartmental models and ABMs can inform policy-makers and support decision processes. For that purpose, it is discussed their relevance for prediction (Sect. 4.1), explanation (Sect. 4.2), and intervention (Sect. 4.3).

## Compartmental models

Epidemiological models have been prominent in the COVID-19 pandemic. Predicting the evolution of the pandemic and informing governments’ decisions for controlling it are the basic demands for mathematical epidemiologists (Jewell et al., 2020). Compartmental models—originally formulated in (Kermack et al., 1927)—are standard tools in this field.

### The SIR model

The simplest compartmental model is *SIR*. It is a set of three ordinary differential equations which try to describe the rate of change in relation to three different compartments in a particular population: Susceptible (*S*), Infected and infectious (*I*), and Recovered and neither able to be infected again nor to spread the disease (*R*). During an epidemic episode some individuals move from *S* to *I*, and then, to *R*. The equations are intended to predict how the number of individuals in each compartment changes as epidemics evolve:

$$\frac{dS}{{dt}} = - \beta S I$$

$$\frac{dI}{{dt}} = \beta S I - \gamma I$$

$$\frac{dR}{{dt}} = \gamma I$$

The parameters *β* and *γ* are the “infectivity rate” and the “recovery rate”, respectively. *β* depends on the number of contacts an infected individual has per time unit (*κ*) and the “transmission rate”, that is, the probability of transmitting the infection to susceptible contacts (*τ*). Then, $\beta = \frac{\kappa \cdot \tau }{N}$. And $\gamma = \frac{1}{D}$, where *D* is the duration of the infection, measured in units of time (days, for instance).

This model makes some assumptions:

- population is closed, so if *N* is the number of members at *t*_0_, for the entire process *S* + *I* + *R* = *N*
- natural births/deaths are not considered
- the outbreak is short lived
- the latent period is null: an individual becomes infectious at the moment she is infected
- no backward transfer is allowed, so recovery equates to lifetime immunity
- even though the disease can be fatal, the recovery rate does not distinguish between recovered or death individuals
- *β* and *γ* are constant (unaffected by age of patients, virus mutations…)
- homogeneous mixing: all infected individuals transmit the disease at rate *β* and the recipient is chosen uniformly at random from the population

According to all this we can plot the epidemic curve (three curves, indeed: one for each compartment).^2^

A central question for predicting the evolution of the epidemic seems to be how many people is infected by an infectious individual for the time she is ill and able to transmit the infection, that is, before her recovery or death. This is *R*_0_, the *basic reproduction number*, which is derived from *R*, the *reproductive rate*, a notion developed by Anderson and May (1982); see also (Heesterbeek, 2002):

$$R = \frac{{S_{0} \cdot \beta }}{\gamma }$$

At the outset of the epidemic just one individual, perhaps a few of them, is infected. If the whole population, except those few ones infected at the beginning, may be infected, and *S*_0_ >  > *I*_0_*,* then *S*_0_/*N* *≈* 1. Given that, since $\beta = \frac{\kappa \cdot \tau }{N}$, we obtain *R*_0_, that is:

$$R_{0} = \frac{ \kappa \cdot \tau }{\gamma } = D \cdot \kappa \cdot \tau$$

*R*_0_ is the average quantity of people who gets infected by an infectious person during the whole period the latter is infectious. It is a good indicator of the near tendency of the epidemic. Thus, if *R*_0_ > 1, *I* exponentially grows; if *R*_0_ < 1, *I* exponentially decreases.

### Pros and cons of compartmental models

Compartmental models have been routinely used for different infectious diseases for a long time. Measles, dengue fever, influenza, HIV, and more recently, the SARS epidemic of 2002, the H1N1 influenza pandemic of 2009, and the Ebola outbreak of 2014, are some examples (Brauer, 2017; May & Anderson, 1987). The equations of compartmental models are not very demanding concerning the amount of input to be implemented. Some basic knowledge about the disease mode of transmission is assumed, of course. Apart from this, cardinals for *N* and *I*_0_, plus estimated values for a few parameters suffice.

This is a good reason to favour compartmental models in some situations. Think of a sudden outbreak of an epidemic and a disease with a non-negligible rate of fatality. Delay in decisions could result in an unacceptable increment of deaths. Here, compartmental models may be, in principle, a fine option. We have previous implementations of them in many different epidemics and the information required for calibrating them is not very extensive when compared to some other modelling options (see below). Certainly, if the disease is unknown, it may be extremely difficult to get reliable information about those parameters at the onset of the epidemic. However, this is an unavoidable constraint not only for compartmental models, but for every prospective strategy.

Nonetheless, compartmental models also have some limitations concerning individual variability, social dimension of the epidemic, unexpected responses to interventions, and side effects of interventions. Let us pause on this.

(1) *Individual variability*. Individuals are initially classified as members of either *S* or *I*, and the focus is on the global rate of change from *S* to *I* and from *I* to *R*. Hence, compartmental models are not very realistic about some traits of individuals that may be highly relevant for a detailed understanding of the human transmission pathways and, consequently, for improving local predictions about the disease spreading. As we noticed before, it is possible to include additional compartments—for the exposed, for infectious who require hospitalization, etc.—to build more complex compartmental models, but that does not change the essentials.

Regarding sources of individual variability, we should distinguish between disease independent host factors—e.g., sex, age, number of contacts, and compliance to public health recommendations—and disease dependent host factors—e.g., susceptibility to disease, transmission rate, and recovery rate. The infectivity rate is a partial effect of social interactions in time and space between individuals, and consequently, to specific habits related to age, environmental conditions… Individual biological response to the infectious agent may introduce variations in the probability of catching the disease, specifically, in the transmission rate *τ* (there are many reasons why a particular individual may be more/less contagious when contact with susceptible people is established), and also in the recovery rate *γ* (notice that we are assuming, firstly, that the period of being infected equates to that of being infectious, and secondly, that this is the same for all individuals in *N*).

Let us consider in detail a fundamental disease independent factor as the number of contacts per time unit (*κ*), usually measured in number of daily contacts. A homogeneous distribution of the population in space is assumed in compartmental models so that contact with every other person per time unit has equal probability. The “mass-action” principle is related to this. According to it, doubling the size of the population implies doubling the number of infections per time unit. But our contacts are much more frequent with those people belonging to smaller subgroups (close friends, family…). The number of contacts also depend on age or activity. Appealing to multiple classes of susceptible and infected individuals and assuming well mixing between these sub-classes with different rates is a lively option. But at the onset of an epidemic there are few individuals infected and the specific pattern of contacts of a typical infectious agent seems relevant. Furthermore, individual peculiarities have been noticed in many epidemics. “Superspreaders”—i.e., individuals who transmit the infection to many susceptible members—have been observed, while some others transmit the infection to very few people.^3^

In order to cope with these difficulties some authors tried to cash out compartmental models in the network language (Brauer et al., 2008; Miller, 2017). Still, when compared with empirical epidemiological data, homogeneous mixing models fit better when the real pattern of contacts involves a highly connected network where many individuals make contact. Again, that may be an unrealistic state of affairs. Besides, they have a tendency to underestimate the amount of initial spread for the transmission and to overestimate it at the final period.

(2) *Social dimension of the epidemic.* Prediction is the main goal for COVID-19 compartmental models. As the proportion of recovered people increases there are fewer susceptible contacts to be infected, so eventually *R*_0_ goes down. The evolution of the epidemic is determined by changes in *R*_0_, but these fluctuations do not depend just on bio-medical factors. Now, according to the parameters included in compartmental models, what strategies could be followed for controlling the epidemic?

- A direct defensive move is vaccination. Vaccinated people reduce the proportion of susceptible individuals among the contacts of an infectious person and, presumably, the number of contagions she provokes while she is ill.

But there are further possible interventions in order to minimize *R*_0_:

(b) Decreasing the duration of the infectious period (*D*).
(c) Reducing the number of individuals’ contacts per time unit $(\kappa$).
(d) Reducing the probability of transmitting the infection in contact with susceptible people (*τ*).

Options (a) and (b) depend on the availability of effective drugs. Unfortunately, there were no effective drug-pharmacological treatments against COVID-19 till the advent of vaccines at the end of 2020. In contrast, (c) and (d), which could be labelled as “non-pharmacological interventions” (NPIs), were available. Isolation and preventive hygienic measures as lockdown and handwashing, respectively, were recommended.

Furthermore, different degrees of severity can be envisaged here. Voluntary isolation of infectious people is a minimum (appealing to personal responsibility) while compulsory quarantine for them and for their close contacts would be a stricter policy. A much more ambitious option to prevent the circulation of the virus is forced isolation at home for all people (without distinguishing between those included in *S*, *I*, and *R*) except for essential workers. In fact, measures applied in different countries greatly differed—compare, for instance, Spain or Italy, where severe lockdown was applied, to Sweden.

The fact is that fluctuations in *R*_0_ are influenced by more or less spontaneous changes in the agents’ behaviour, but they may also be externally provoked by new legal provisions (closing schools, for instance). Accordingly, social attitudes and political decisions, in addition to bio-medical factors, are decisive to predict the course of the epidemic. Some authors even insisted that changes in *R*_0_ during the COVID-19 pandemic are not governed by “natural” laws (Boumans, 2021). If that means that conditions where natural laws operate change as a result of policy-makers’ decisions, we agree on that. Changes in *R*_0_ cannot be fully explained resorting exclusively to those laws. And this adds a significant degree of uncertainty on the evolution of the pandemic.^4^

In favour of compartmental models it should be stressed, however, that they have been used not only at the “null-action” setting, i.e., when no public global intervention has been tried, but also to assess the potential effects of measures as quarantine, social distancing, etc. Certainly, NPIs are aimed at reducing the values of either *κ* or *τ* by favouring or disallowing certain behaviours and controlling the conditions for interaction between citizens. Insofar as NPIs have an impact on those endogenous factors highlighted by compartmental models, it would be unfair to claim that the social dimension of COVID-19 pandemic is completely disregarded by those models.^5^

Regrettably, the resources of compartmental models to address the social dimension of the pandemic are limited. Changes in patterns of social interaction brought about by NPIs can only be grossly conjectured. Those patterns, like health policies, should properly be understood as exogenous variables in those models. In addition to this, social conditions independent of the implemented policies could significantly erode the effectiveness of such measures in slowing the spread of the epidemic. Think about specific behaviours resulting from low-income effects (necessity of going out for work violating quarantine, avoiding use of face masks because of their price…) or differences in health services equipment/qualification among countries, regions, cities, or neighbourhoods.

(3) *Unexpected responses to interventions.* Predictions about the evolution of a contagious disease like COVID-19 cannot overlook changes in human attitudes and behaviour. Of course, in order to predict the effectiveness of social policies some expected effects on behaviour are considered by compartmental models. Severe lock-down accompanied with high fines guarantees that many outdoor activities will not be practiced by the vast majority of people. But there may be effects on the individual response that: (1) are more difficult to predict, (2) may have a substantial influence—positive or negative—in the course of the epidemic statistics at least in particular locations, age cohorts, etc., and (3) can hardly be accommodated in compartmental models.

Among those consequences, we could distinguish:

*Spill-over effects*: unexpected effects in the “control” population (that is, the part of the population where the intervention does not apply) as a consequence of an intervention in the target population. Forced quarantine just for infectious people, for instance, may make their close relatives and neighbours more careful with hygiene preventive measures.

*Looping effects*: unexpected changes in the behaviour of the target population as a consequence of an intervention, which could have a non-negligible influence, be it positive or negative, on its effectiveness. The more extended period of closure for businesses where alcoholic beverages are allowed, the higher increase in multitudinous private parties where security measures are not enforced. This may have a differential impact (i.e., a remarkable increase of infected people) among those population groups who attend to these events but, perhaps, a minimal impact on some other groups.

Even though asking models for very accurate predictions about this sort of effects could be unrealistic, a broad anticipation of these differential impacts allows for devising specific counterbalancing policies. But compartmental models have no slot in their input for behavioural considerations like these.

(4) *Side effects of interventions*. In addition to those individual responses that could have an indirect influence on the effectiveness of the implemented policies, there are further untargeted effects that are out of reach of compartmental models. When confronted to the decision to extend severe lockdowns, for instance, a cost–benefit calculation should take into account that long lockdown periods may have an impact on demand for mental health care. Likewise, it has been noticed that closing schools is more disadvantageous for those children who have no easy access to new technologies at home. Then, it is not a surprising fact that a pandemic like that of COVID-19 has effects in areas as diverse as mental health, education, job market… The point is anticipating and estimating—with a moderate degree or reliability at least—these partly expected but unintended consequences of NPIs. Compartmental models, however, are silent about them.

## Agent-based models

Although compartmental models are standard tools, epidemiologists also appeal to other kinds of models to predict the evolution of epidemics and anticipate the (plausible) effects of certain interventions. Among those alternative approaches, ABMs occupy a prominent place. Since its emergence in the second half of the twentieth century, they have achieved great relevance in fields such as sociology (Macy & Flache, 2009), political science (de Marchi & Page, 2014), social psychology (Smith & Conrey, 2007), immunology (Bauer et al., 2009), and epidemiology (Auchincloss & Diez Roux, 2008). In this section, we will characterise ABMs and discuss their main capabilities and limitations in epidemic scenarios.

### Dynamics of behaviour

An ABM can be an artificial society (Epstein & Axtell, 1996). ABMs are computer simulations of autonomous agents that interact according to a set of rules and within a specific environment (Epstein, 2006). Individual agents, which are the theoretical starting point of any ABM, are represented as specific software objects; they are not aggregated into homogeneous populations. Each individual agent is characterised by a vector of attributes, usually including spatial location (de Marchi & Page, 2014). Some other attributes could be gender, political affinity, job… So, the set of attributes is:

$$\left\{ {X_{1} , X_{2} , \ldots X_{A} } \right\}\quad X_{i} = \left\{ {x_{i1} ,x_{i2} ,x_{i3} , \ldots } \right\}$$

Models are instantiated by assigning particular values—$x_{i1} ,x_{i2} ,x_{i3} , \ldots$—to agents for each attribute. A great flexibility is allowed regarding the individual agents included in the model. They can be few or millions, identical or highly heterogeneous, etc. Furthermore, attributes can be fixed, and remain invariable during the entire simulation, or mutable and susceptible to change. For time-indexed attributes, the state of agent *j* at a time *t* is:

$$s_{j}^{t} = \left( {x_{j1}^{t} , x_{j2}^{t} , \ldots x_{jA}^{t} } \right)\quad x_{ji }^{t} \in X_{i}$$

Provided that the number of agents is *N*, the configuration of the model at *t* encompasses all the agents’ states at *t*:

$$S^{t} = \left\{ {s_{1}^{t} , s_{2}^{t} , \ldots s_{N}^{t} } \right\} \left( {x_{j1} , x_{j2} , \ldots x_{jA} } \right)\quad x_{ji} \in X_{i}$$

Individual agents interact among them and with the environment on the basis of behavioural rules, which can also be fixed or mutable. Those rules can be the same for all the agents or vary among them, allowing heterogeneous behavioural patterns. They guide individuals’ behaviour according to the input they receive from environment. That input may refer to past configurations of the environment, current configurations of the environment, or potential actions of other agents. Because of the responsive nature of those rules, the behaviour of agents is often considered as adaptive (de Marchi & Page, 2014). Nevertheless, it should be noticed that, in most ABMs, agents have limited cognitive capacities and limited information, which is often restricted to the nearby environment (Parker & Epstein, 2011). Accordingly, given a particular (past, current, or future) configuration of the environment, they do not always make optimal choices.

In ABMs, the autonomous and interdependent behaviour of individual agents aggregates and results in (often informative) system-level outcomes (e.g., economic inequality among neighbourhoods). Those outputs are usually understood as emergent or bottom-up (Epstein, 2006; Macy & Flache, 2009). They can be highly unexpected even when agents’ attributes and behavioural rules are well known. ABMs pay special attention to *how* individual behaviour results in system-level outcomes (de Marchi & Page, 2014). They aim to identify and explore the link between some individuals’ traits and behaviours, and certain aggregate patterns. Hence, unlike compartmental models, ABMs are not exclusively focused on the aggregate output, but also on the dynamics of the individuals’ behaviour.

Concerning the degree of detail, ABMs vary significantly in their complexity and their resemblance to the target real population. They can range from simple and unrealistic models, such as the well-known segregation model developed by Schelling (1971), to complex and empirically calibrated models. The Global-Scale Agent Model (GSAM), for instance, includes more than 6 billion individual agents that are modelled on the basis of available data (Parker & Epstein, 2011). The appropriate level of complexity and realism depends on the purpose of the model (Boero & Squazzoni, 2005; Bruch & Atwell, 2015; de Marchi & Page, 2014).

During the COVID-19 pandemic, several ABMs were built to study and predict the viral spread and the plausible effect of diverse interventions. In this sense, for example, Nicolas Hoertel et al. (2020) developed a stochastic ABM of the epidemic in France. The main purpose of their research, which was conducted in the spring of 2020, was to study the consequences of lifting the first nationwide lockdown and evaluate the efficacy of diverse NPIs to avoid a second epidemic peak and lockdown. They examined the potential impact of post-lockdown measures on cumulative disease incidence and mortality, and on ICU-bed occupancy. Three of them were discussed: physical distancing, mask-wearing, and shielding those who are the most vulnerable to severe COVID-19 infection.

The ABM developed by Hoertel and collaborators consisted of a realistic synthetic population (500.000 agents), a social contact network among individual agents (including close/prolonged, less-frequent/less-prolonged, and brief contacts), and a disease model. The model included 194 parameters that were considered potentially relevant. Among them, 140 parameters referred to individuals’ properties (e.g., age), 33 to social contacts (e.g., employment rate), and 21 to SARS-CoV-2 characteristics (e.g., incubation time). Most of those parameters were calibrated on the basis of available data. The main references for calibration were data provided by the French National Statistical Institute and Santé Publique France for individuals’ properties, information provided by previous studies for social contacts, and data provided by the Pasteur Institute and the Imperial College London for SARS-CoV-2 characteristics.

Nevertheless, at the first stages of the pandemic, when the model was built, few data were available about basic aspects of SARS-CoV-2 transmission. In particular, the proportion of undiagnosed cases and the risk of contamination were almost entirely unknown. Those parameters were estimated by comparing model predictions about previous stages of the epidemic with the observed data. For that comparison, data provided by the Pasteur Institute and Santé Publique France were taken as reference. Furthermore, sensitivity analyses were conducted in order to ensure that deviations in the estimation of those parameters would have little impact on the results. In those sensitive analyses, several versions of the ABM, which only differed in the value of the uncertain parameter, were run and their outputs were compared. In fact, in order to support the robustness of the model, sensitivity analyses (with variations of ± 20%) of each individual parameter were conducted.

In the empirical calibration of the model, not only parameters about SARS-CoV-2 transmission were problematic. There was also scarce information about important aspects of social contacts. There were no data about parameters such as number of shopping trips (per week), frequency of meeting friends (per week), or number of close encounters per event participation. The estimated value of those parameters was based on assumptions about social behaviour. Furthermore, as it has been noted, sensitive analyses were also conducted regarding all parameters.

The ABM developed by Hoertel and collaborators anticipated that physical distancing and mask-wearing would be effective in slowing the epidemic and reducing mortality, but they would hardly be sufficient to prevent overwhelming ICUs and a second lockdown. However, according to that model, complementing physical distancing and mask-wearing with shielding of vulnerable people (for a period of 38 weeks) would result in better outcomes, including lower mortality and an adequate ICUs occupancy. Nonetheless, they claim that, in both scenarios, benefits would be substantially reduced if the measures were not adopted by most people or were not maintained for a sufficiently long period.

### The potential of ABMs

Advocates of ABMs have pointed out several capabilities and strengths of that kind of models (see, for instance, Auchincloss & Diez Roux, 2008; de Marchi & Page, 2014; Macy & Flache, 2009). They are able to model dynamic processes, link individual behaviour and population-level outcomes, explore neighbourhoods of models, include (geographical and social) space, etc. Nevertheless, there are four traits of ABMs especially relevant in epidemic or pandemic scenarios: heterogeneity of agents, variety of interventions, dynamic response, and identification of side effects. Let us consider them in some detail.

(1) *Heterogeneity of agents*. In ABMs, each individual agent is represented as a specific software object and characterised by a particular vector of attributes. Furthermore, behavioural rules are individually assigned to each particular agent. Individual agents can differ in beliefs, location, information, preferences, ability, learning rules, etc. Consequently, it is possible to build artificial societies that incorporate the heterogeneity and diversity of the target population (Auchincloss & Diez Roux, 2008; de Marchi & Page, 2014). Following epidemiological and biological databases, ABMs can include those aspects of individuals relevant for the spread of the disease. Those aspects can be either disease independent host factors (e.g., sex) or disease dependent host factors (e.g., susceptibility to disease). In order to illustrate that point, recall the ABM developed in Hoertel et al. (2020). In that model, individual agents diverge in many aspects that are considered relevant for the spread of the SARS-CoV-2. They differ in individual traits such as age or pre-existing conditions, and also in social aspects such as number of colleagues at work or use of public transport. ABMs create artificial societies whose individual components resemble the real population of interest and take into account the influence of (non-homogeneously distributed) relevant aspects. This possibility of building heterogeneous populations is aptly appreciated to enhance ABMs accuracy in prediction.

(2) *Variety of interventions*. The theoretical starting point of ABMs are individual agents, which are individually characterised. And, as it has been noted, many and diverse parameters can be incorporated to characterise individual agents and environments. Those parameters can refer to biological traits, cognitive capacities, social topologies, etc. This flexible and bottom-up nature allows ABMs to contemplate, study, and evaluate diverse interventions (Manzo, 2020). Although, as in the case of compartmental models, health policies are exogenous to them, ABMs can take into account a great array of interventions. They can explore interventions in any attribute or behavioural rule of any individual agent or type of agents, and also in any parameter of the environment. The interventions can range from very general interventions that address the whole population to fine-grained interventions targeted to a particular kind of agents. For instance, an ABM can simulate a 25% reduction in the use of public transport by agents who have close contact with agents with pre-existing conditions that make them particularly vulnerable to the disease. The possibility of considering diverse kinds of interventions has been important for the evaluation of NPIs to mitigate the COVID-19 pandemic. For example, the ABM of the epidemic in Ontario developed by Naimark et al. (2021) takes into account both community-based NPIs (e.g., restricting gatherings) and non-community-based NPIs (e.g., closing schools), and compares their influence in the number of infections. The main aim of the model is to study if non-community-based NPIs, which have many undesired effects, can be avoided.

(3) *Dynamic response*. In ABMs, agents interact guided by behavioural rules which may differ among them. Those rules, as it has been noted, guide their behaviour on the bases of the input received from the environment. Given this adaptive and dynamic aspect, ABMs are able to explore changes in people’s behaviour as result of the introduction of a particular intervention (Bruch & Atwell, 2015). In that kind of models, as long as the intervention results on a change in (some of) the included parameters, individual agents will act in response to it. The specific response will depend on both the changes produced and the behavioural rules. The input encompassed by behavioural rules can include traits of the own agent, of other agents, and of the environment. The flexibility of behavioural rules allows ABMs to incorporate both spill-over effects (i.e., untargeted effects on the population not targeted by the intervention) and looping effects (i.e., untargeted effects on the population targeted by the intervention).

(4) *Identification of side effects*. ABMs may include multiple processes, which involve different domains, in the same instantiation. Individual agents are endowed with very diverse kinds of attributes (psychological, biological, economic, etc.) and they interact across those multiple domains. For example, a viral infection can bring about health, occupational, and psychological effects on a particular agent. And these changes will probably modify her particular pattern of interactions with other individuals in each of those domains. The capacity of simultaneously accounting for diverse domains and processes places ABMs in a privileged position to identify potential side effects of interventions, even although those effects refer to domains not targeted by the intervention (e.g., cross-domain spill-over). Furthermore, given the adaptive and dynamic nature of agents’ behaviour, ABMs can also incorporate feedbacks from one domain to other. In fact, ABMs have been used to identify and weight the plausible side effects of NPIs in the current pandemic. For example, Kano et al. (2021) developed a simple ABM to explore the relationship between the spread of COVID-19 and economic activities. In that model, both health (e.g., infection) and economic traits (e.g., employment) of agents are considered, and special attention is paid to how they are related. For example, the authors analysed how the amount of previous savings relates with the probability of being infected.

### The limitations of ABMs

As it has been noted, ABMs can vary significantly in their complexity and realism. They can range from simple and unrealistic models to complex and empirically calibrated models. The adequate degree of complexity and realism depends on the goals of the model. For instance, for pure theoretical purposes such as illustrating new intuitions or ideas, abstract and unrealistic ABMs are the most appropriate (Boero & Squazzoni, 2005).

In epidemics, as we have seen, the main role of ABMs is to assess the effect of particular factors or interventions in specific populations. For that purpose, it is usually considered that complex and realistic models are required. That sort of ABMs are often known as “high-fidelity” (de Marchi & Page, 2014), “high-dimensional realism” (Bruch & Atwell, 2015), or “case-based” (Boero & Squazzoni, 2005) models. In those high-fidelity ABMs, model components must be empirically calibrated. Available empirical data and insights from diverse fields of research (e.g., social psychology) must be taken as reference for deciding which parameters include and fix their value. Yet, high-fidelity ABMs face important difficulties that may undermine their assets in epidemics.

First, the relevance and accuracy of high-fidelity ABMs depend on their empirical adequacy (Auchincloss & Diez Roux, 2008; Jewell et al., 2020). Those complex ABMs involve many parameters that must be empirically calibrated. Unfortunately, it is often the case that available data about some of those parameters is scarce (or unreliable) and, consequently, adequate empirical calibration is hardly achievable. That scarcity of data is an outstanding source of uncertainty—i.e., input uncertainty—and may undermine the predictions made by the model (Bauer et al., 2009; Bruch & Atwell, 2015). It should be added that empirical calibration is especially problematic in the early stages of epidemics of new diseases. At the beginning of the COVID-19 episode, little information was available about essential aspects of the disease such as incubation period, protective immunity, or proportion of asymptomatic (Holmdahl & Buckee, 2020). Furthermore, how people would behave in a global pandemic scenario was difficult to anticipate.

In order to calibrate parameters in non-ideal conditions, different strategies have been developed (Bruch & Atwell, 2015). For example, in the model developed by Hoertel and collaborators (see Sect. 3.1), the uncertain parameters are estimated by comparing the model-predicted data with the observed data. Nonetheless, in complex ABMs, those strategies are hardly unproblematic. In those models, the parameters space is highly expanded and exploring it results complicated. As a consequence, estimation of unknown parameters is always tentative. For example, there may be several (known or unknown) combinations of values that result in the observed output.

Second, in high-fidelity ABMs, many interactions, dynamic processes, and outcomes take place (Auchincloss & Diez Roux, 2008; Bauer et al., 2009; Macy & Flache, 2009). They generate very complex networks of micro-micro and micro–macro relations. As a result, interpreting the model becomes extremely difficult. It may be impossible to identify and understand the causal processes that underlie the detected system-level outcomes. ABMs may, somewhat paradoxically, become opaque. This opacity makes it difficult to assess the role played by a specific factor or intervention, that is, to understand *how* and *to what extent* it has contributed to a specific system-level output.^6^ Moreover, when unexpected or undesired effects are identified, tracing their history and identifying their causes is hardly possible.

And third, high-fidelity ABMs can be seen as case-based or ad-hoc models (Boero & Squazzoni, 2005). They take into account many specific aspects of the real population of interest, which characterise and distinguish it from other populations. That specificity is important in order to make accurate predictions. Nevertheless, due to the case-based nature of high-fidelity ABMs, their results and the conclusions drawn from them are hardly generalizable. Furthermore, given the complexity of those models, it is very difficult to anticipate which deviations in the examined parameters would have a significant impact in the output.

Of course, in principle, it is possible to recalibrate the model for each population of interest and run multiple simulations. Alagoz et al. (2020), for instance, recalibrated an ABM of the COVID-19 epidemic to analyse the effect of several aspects of social distancing (adherence, timing of implementation, and timing of easing) in diverse populations (Dane County, Milwaukee Metropolitan Area, and New York City). But this kind of approach is not unproblematic. While material resources (including time, economic funds, and workforce) are often scarce, target populations may be many and very diverse. Therefore, it is not always possible to build a specific high-fidelity ABM for each population.

## Models, evidence, and decision-making

According to the evidence-based paradigm, medical practice and policy interventions should be guided by evidence. Evidence is evaluated and weighted according to hierarchies, which rank kinds of methods according to their potential to suffer from systematic bias (Howick, 2011; Karanicolas et al., 2008). In hierarchies of evidence, Randomized Controlled Trials (RCTs) and meta-analysis of RCTs are typically at the top, followed by observational studies (e.g., cohort studies). The bottom is often reserved to case studies, expert opinion, and mechanism-based reasoning. Among the most prominent hierarchies are those developed by the Oxford Centre for Evidence-Based Medicine, the National Institute for Health and Care Excellence (UK), and the Scottish Intercollegiate Guidelines Network.

In the last decades, the evidence-based paradigm has gained great popularity in policy-making. In areas such as development economics, it has become the standard approach (Olken, 2020). The evidence-based paradigm has shaped research teams, funding programs, evaluation parameters, etc.

Nonetheless, in certain contexts, high-quality evidence (e.g., RCTs) is not available and other information must be taken into account. In those scenarios, there is a potential role for scientific models. This is the case when coping with a new infectious disease, and COVID-19 was not an exception, at least during the first stages of the pandemic (Pearson, 2021). But there seems to be some additional reasons for that unavailability of non-fragmentary evidence: urgency, practical limitations, and ethical concerns. First, given the rapid spread and lethality of the COVID-19, a delay in the development and implementation of treatments and interventions until the realisation of high-quality and time-demanding RCTs was undesirable. It should be noted that the rush for obtaining RCT-based evidence often results in poor-quality studies (e.g., small sample, absence of control group, etc.). Second, because of their methodological basis, RCTs were unable to provide us with evidence about certain relevant causal hypotheses. Interventions such as confining a territory or closing borders between countries can hardly be evaluated by them. And third, due to the hazards of COVID-19 and available background knowledge, it was ethically objectionable to test certain causal hypotheses by means of RCTs. For example, RCTs about side effects of ibuprofen in COVID-19 patients would have been an inadmissible risk for individuals in the treatment group.

The fact is that epidemiological models, given the scarcity of high-quality evidence, have played an important role in the COVID-19 pandemic. And even the staunchest advocates of high-quality evidence have claimed that taking models into account was a sensible choice (see, for instance, Ioannidis, 2020). But even though it is generally accepted that epidemiological models may be a valuable resource, a fundamental question remains open: which kinds of models should be employed? That is, on account of their basic properties, should certain kind of models be favoured in (certain scenarios of) policy-making? Since contributing to the development of health/social policies partly depends on models’ predictions and on the understanding afforded by them, their effective contribution to interventions should not be considered separately from their predictive and explanatory merits. On the basis of the previous discussion, we will compare compartmental models and ABMs with respect to those three goals.

### Prediction

Models must be implemented with some empirical data to generate more or less specific predictions. In principle, the more uncertain those data, the riskier the predictions. However, unavailability of comprehensive data was a salient aspect at the COVID-19 pandemic outbreak. Although our knowledge about the virus (SARS-CoV-2) and the disease gradually increased through the last year, initially very few details were known about them. There were just very limited statistical surveys and tentative hypothesis about primary routes of transmission, immunity after recovery, reproductive rate, potential treatments, etc. A negative effect on the predictive performance of models was to be expected. Despite all this, compartmental models were developed. Those early compartmental models were indeed the main guide for political decision-making in the beginning of the epidemic. It is worth stressing here that the input required for those models was just about a few parameters. In contrast, ABMs, and particularly the high-fidelity ones, require a lot of empirical input. As Kathleen O’ Reilly, an epidemiologist at London School of Hygiene and Tropical Medicine, claimed: “These very specific models are extremely data hungry” (Adam, 2020, p. 317). Attributes and behavioural rules of each individual agent, in addition to the disease model, must be empirically calibrated. As a result, a huge amount of information about the modelled population is required. Even though sensitivity analyses can be conducted to assess the potential impact of inaccurate estimations, it has been noticed that those procedures are hardly unproblematic (see Sect. 3.2). Furthermore, given the dynamic nature of ABMs, an inaccurate parameter may have a huge impact on the system-level outcome.

Then, there is a remarkable difference between compartmental models and ABMs in their respective demands on data. Insofar as uncertainty about this information may jeopardize prediction, at the beginning of the COVID-19 pandemic compartmental models could be run with more confidence. During the first wave of the pandemic, the main goal was to predict the number of COVID-19 cases and, especially, the number of deaths with and without the introduction of NPIs. Compartmental models were decisive for that purpose (see Sect. 2). They provided early predictions that warned us about the dangers of that new disease and encouraged the introduction of some NPIs.

Let us now put aside the quality and quantity of data by assuming that we have a reliable and fairly comprehensive stock of empirical information. Differences in predictive performance between both sorts of models are not erased, anyway. Concerning model-based predictions, a well-known distinction differentiates between unconditional predictions (*forecasts*) and conditional predictions (*projections*) (Fuller, 2021; Schroeder, 2021). The former predict how the system under study will actually be in the future. Projections, on the other hand, predict how the system would be/evolve given certain conditions. Projections are usually contemplated just as hypothetical scenarios, conditioned to some assumptions. It should be noted that, given their hypothetical nature, they are especially difficult to assess since it is often unfeasible to contrast them with empirical data.

In order to evaluate and judge a particular epidemiological model, empirical confirmation should be taken as a crucial guidance. The accuracy of the model's is essential for the purposes for which models are used in policy-making. Nonetheless, our discussion here is not about the predictive success of any particular model, but on the fundamental traits of compartmental models compared to ABMs, that is, on how the way they are built and their respective capacities and limitations are related with the demands and needs of policy-making.^7^ And from this general point of view some morals can be obtained. Thus, when faced with local scenarios, compartmental models have important predictive limitations. Those limitations are mainly related to their difficulties for taking into account individual variability and (certain aspects) of social behaviour. Compartmental models follow the evolution of epidemics from a population-level point of view, in which the agents that constitute the population are not individuated but merged, producing average types for each compartment. Consequently, particularities of individual agents (e.g., pre-existing conditions) and their interactions (e.g., number and kind of contacts) can hardly be tackled.^8^ ABMs seem to be in a better position regarding local-oriented prediction. They are specifically devised to take into account individual heterogeneity and particular social behaviour. In ABMs, modellers start specifying the traits and rules of behaviour of each individual agent and then run the simulation to see the resulting outcome.

### Explanation

Although prediction was a central goal, especially in the first stages of the COVID-19 pandemic, models were also used for explanatory purposes. They aimed to provide information about which factors increased the speed of contagion, how the virus spreads among a population, etc. That information is notably valuable for policy-makers since it points at those aspects that should be targeted to achieve the desired effect, i.e., controlling the epidemic.

Compartmental models, in particular, give a valuable account of the dynamics of disease spread. They are specially fit to identify the basic parameters underlying the evolution of an epidemic from a general perspective. That means that compartmental models may be successful in explaining how the disease evolves through populations. To certain extent, these are “black-box explanations” whose simplifying assumptions can be accepted insofar as what we are concerned about is the rate of transition between the different compartments. In this context, the scope of decisions is significantly constrained by the very same information provided by the model. They must be targeted to questions as how to minimize the whole number of susceptible individuals, the rate transition from susceptible to exposed, from exposed to infectious, and so on. Then, interventions aimed at this population-level can be reasonably informed by the explanatory account afforded by compartmental models. In this sense, compartmental models “can only lead to one type of intervention, i.e., interventions that indifferently concern large subsets of the population or even the overall population” (Manzo, 2020, p. 33). The point is that selective interventions to operate on particular subgroups in the population demand further information that can hardly be provided by compartmental models.

ABMs, in contrast, open the black box by specifying *how* certain interactions among individual agents result in the identified system-level output. They provide information about the internal workings responsible for the output. In the methodological literature, especially in social science, ABMs are often associated with the development of mechanism-based explanations (see, for instance, Hedström & Ylikoski, 2010; Macy & Flache, 2009; Manzo, 2010). In order to specify the mechanism responsible for the phenomenon of interest—i.e., identifying its component entities and activities—building an ABM able to generate that phenomenon is considered a crucial step.^9^ Mechanism-based explanations, in principle, can be of great value for policy-making. They specify the causal processes responsible for the phenomenon of interest and, consequently, provide valuable information about the relevant aspects that should be addressed by interventions.

Nonetheless, fine-grained ABMs’ have some disadvantages. As the number of agents and the array of characteristics assigned to them grow, models become more complex. While exploration of different scenarios for compartmental models is restricted to a limited number of parameters, the situation is much more complicated for complex ABMs. The network of interactions that links individual agents and system-level outcomes trough different temporal stages may gradually become more elusive. Thus, it can be very complicated for researchers to discern the causal chain responsible for the resulting outcome.

### Intervention

Policy-making is not only informed by the evidence listed in and assessed by hierarchies. As it has been argued in previous sections, models were used to anticipate the spread of the SARS-CoV-2, test NPIs, identify plausible side effects, etc. This role was particularly salient under the extraordinary conditions triggered by the pandemic. But it is worth emphasizing here that models may also be a valuable source of information, which can complement available evidence, in less pressing situations.

First of all, it must be acknowledged that there is a feed-back between policy-makers and modellers (Manheim et al., 2016). During the COVID-19 pandemic, the former demanded prospective research to scientists in order to anticipate the spread of diseases, the cost in human lives, the capacity of the health system to cope with increasing demands, the effects of more or less strict measures… Modellers gave predictions and tested the options—in virtual scenarios, certainly—considered by policy-makers (McBryde et al., 2020). Models also suggested new interventions and qualifications of those measures initially implemented.

A great advantage of modelling is precisely the possibility to explore counterfactual settings which can hardly be implemented in practice. Virtual exploration is one source of information about those scenarios for policy-makers. Modelling may help decision-makers to anticipate future states of the populations of interest, and to develop and test interventions aimed to produce specific effects in those populations. Nevertheless, it should be noted that, in order to be helpful for policy-making, modellers must inform about the capabilities and limitations of implemented models (Manheim et al., 2016).

Turning now to the comparison between compartmental models and ABMs, how do they score regarding intervention? Policy-making, unlike political science and other theoretical fields, is usually focused on particular contexts and populations. Its main aim is to develop, assess, and implement policy interventions for obtaining certain desired effects (or, alternatively, for avoiding certain undesired effects) in particular contexts. According to the aforementioned comments, if we are interested in very detailed predictions on possible NPIs, ABMs should be favoured against compartmental models, in principle, provided that the empirical input is fairly good at least. Still, compartmental models may be fully adequate in different conditions.

Summing up, the predictive performance of models decisively depends on the availability of good-quality data and also on the scope of prediction. In principle, ABMs are more adequate for local predictions while compartmental models fare better when the goal is getting a general view on the evolution of the epidemic and on the effects of policies. Turning to explanation, compartmental models are interested in explaining the dynamics of the epidemic from a population-level perspective while ABMs may be the appropriate modelling tool for explaining how individual behaviour and characteristics affect disease transmission. Finally, when intervention comes into play, particular demands of policy-makers should be crucial for favouring one or other sort of models.

Correspondingly, we think that it would be very simplistic to affirm that one or other sort of models is intrinsically better. Model-building is constrained by the available resources for building them and the policy-makers demands. Their appropriateness should be assessed, then, in relation to those factors.

## Conclusion

In the COVID-19 pandemic, where high-quality sources of evidence were not initially available and the need for public-health decisions was urgent, epidemiological models have played a central role. They have been one of the main sources of information and a compass for policy decision-making. Regarding theory-driven epidemiological models, both compartmental models and ABMs held, and still hold today, a prominent position. Nonetheless, they can also be a valuable resource in less adverse scenarios. Because of their contrasting characteristics, compartmental models and ABMs (should) play different roles in decision-making. In principle, compartmental models are useful for general predictions about the spread of a disease, particularly when empirical information is scarce. Contrarily, ABMs are more adequate for local and specific predictions, although they require more empirical input. Since ABMs incorporate the detailed effects of human differences in traits and behaviour, they can also be fruitful to discern the mechanisms responsible for the spread of the disease. Compartmental models, on the other hand, explain the dynamics of the disease spread from a population-level perspective. Finally, regarding intervention, compartmental models may be helpful for developing and testing interventions that concern large subsets of the population (or the whole population), while ABMs are more promising, in principle, when fine-grained interventions are required.

We think that our previous discussion on the assets and limits of those kinds of models throws some light on what we can expect from them and, consequently, may significantly contribute to modelling in the current COVID-19 pandemic and also in similar epidemiological episodes. A better understanding of epidemiological models allows us to use them more appropriately. As we have argued, useful modelling requires to consider the particularities of the context of interest and how the diverse kinds of models cope with them. In this sense, two aspects of the context are particularly important and should not be overlooked: the aim of the models and the available empirical data.

Certainly, relevant aspects of the context have not remained constant during the whole COVID-19 pandemic but have changed over time. In the outbreak of the pandemic, epidemiological models were focused primarily on the expected deaths in absence of intervention and the potential effect of population-level NPIs. Subsequently, nonetheless, it has been stressed the importance of considering the economic, social, and psychological aspects of the pandemic (Naz et al., 2021). Many epidemiological models are now demanded to deal with the effects of both the disease and the public health interventions in those areas. Regarding the availability of reliable data, the first stages of the COVID-19 pandemic were characterised by plural uncertainty since very little was known about the mechanisms of transmission of SARS-CoV-2, the effects of considered NPIs, and the social response to them (Ongaro, 2021). Essential questions still remain unanswered (e.g., the immunity term after recovery/vaccination), but our knowledge about basic biological and social aspects of the spread of the disease has significantly increased, allowing for better empirically calibrated models.

Those changes in significant aspects of the context force us to rethink and re-evaluate our modelling practices. Thus, given the context-dependence of epidemiological modelling and the non-stable nature of the context, we think that favouring one or other sort of models in a particular situation cannot be justified only by appealing to their respective technicalities. While the latter are important, contextual factors as the peculiarities of the process to be modelled and the policy-makers’ goals and expectations must be considered to arrive at a reasonable cost–benefit decision.

These considerations suggest a further difference concerning legitimate sources of information in public-health policy. Standard hierarchies of evidence rank several procedures (RCTs, cohort studies…) according to a general characterization of them and to their potential for avoiding biases. Putting aside the question whether models can be considered as providing evidence in the full sense—an issue that goes beyond the scope of this paper—it is clear that they do provide information that may be valuable for decision-making. Context-dependence, however, makes highly complicated to adopt a general approach that ranks kinds of models concerning the alleged quality of the information obtained through them. On our view, comparison between particular models should be decided on a case-by-case strategy. Considering the relevance—i.e., their difference-making potential—of the factors included in the models would be crucial for that comparison (Maziarz & Zach, 2021).

We have tried to assess epidemiological theoretical modelling concerning prediction, explanation, and intervention. It is noteworthy, however, to underline that intervention here is always targeted at more or less extensive groups. Our discussion highlights, then, a distinctive peculiarity of epidemiology when compared to other fields in medicine, that is, that intervention in epidemiology is, properly speaking, social intervention. Epidemiological intervention is unavoidably constrained by social conditions and its deployment pursues supra-individual effects. We hope, finally, that this paper will modestly contribute to a better understanding of those intertwined dimensions—prediction, explanation, and social intervention—involved in epidemiological modelling.

## Data Availability

Not applicable.

## Footnotes

1 A further option that will not be discussed here are “data-driven models”, also labelled as “curve-fitting approaches” or “statistical models” (Fuller, 2021; Manheim et al., 2016). Regarding the COVID-19 episode, the IHME model of the Institute for Health Metrics and Evaluation (University of Washington) is probably the most famous among them.

2 Two further basic options are *SIS* and *SEIR*. The former does not assume total immunity after infection, while the latter adds a new compartment for exposed individuals. For a survey of different versions of compartmental models see Brauer et al. (2008) chaps. 2 and 5.

3 Compartmental models may include some details about individuals. Report 12 of the Imperial College London builds a *SIR* model to predict the final epidemic size and an alternative *SEIR* to model the epidemic’s demand of healthcare. This *SEIR* model adds more compartments by distinguishing between mild infections and those which require hospitalization (with/without requiring ICU beds). Both models are “age-structured” models: the demographic structure of the population is implemented and the rates of contact between different individuals are estimated across different age groups (Walker et al., 2020). For local predictions, anyway, this may not be enough.

4 Boumans infers from that a stronger claim: “This not only makes the curve erratic but its future development unpredictable.” (Boumans, 2021, p. 14). This conclusion should be qualified. Conditions for transmission may change due to political decisions but this does not imply that the evolution of the curve is unpredictable. After all, the indirect effects of those decisions on *R*_0_ may be anticipated to some extent. The drastic reduction of the value of *κ* as a result of severe confinement, for instance, is foreseeable so that its effects on the shape of the curve can also be approximately estimated.

5 For instance, the compartmental model deployed in McCabe et al. (2021) estimates the potential effects of different lockdown scenarios in ICU resources in France, Germany, and Italy.

6 Horner and Symons (2020) analyse the public code archives of the microsimulation model (CovidSim) developed by the Imperial College COVID-19 Response Team. It should be recalled that the tables included in the famous Report 9 issued at March 2020 (Ferguson et al., 2020)—a determinant in shifting the British Government position to favour stricter measures to confront the pandemic—were generated by that model. After examining the implementation phase for it, they conclude: “A simulator typically requires that the user enter input values for the parameters that are relevant to the model underlying the simulation […] The high number of parameters in this simulator and its resulting complexity cannot be avoided at some level if the model is to assess the effects of even the intervention regimes that have been deployed by various countries. As a result, [it] is unavoidably more difficult to comprehend, correctly use, calibrate, and maintain than lower-fidelity epidemiological models as SIR.” (Horner & Symons, 2020, p. 18).

7 In the COVID-19 pandemic, especially in its early stages, the empirical reliability of epidemiological models was below expectations. Readers interested in this question may look at the comparative—and updated—report provided in (Wynants et al., 2020).

8 Bansal et al. (2007) discusses several alternatives for including detailed information—through nets—in compartmental models. It is concluded that “[w]hen contacts are heterogeneous, it thus makes sense to consider the growing toolkit of tractable network based methods that explicitly consider individual-level contact patterns and can predict the changing structure of a contact network as disease spreads through it.” (Bansal et al., 2007, p. 889).

9 It should be noted that developing a generative sufficient ABM is hardly enough for explaining a phenomenon (Macy & Flache, 2009). There may be alternative specifications able to produce the same output, the involved assumptions about individual agents may be highly implausible, or the underlying process may be too obscure. In order to provide a satisfactory mechanism-based explanation, it also seems necessary to specify the generative process that results in the output of interest (León-Medina, 2017).
